# Restoring tides to reduce methane emissions in impounded wetlands: A new and potent Blue Carbon climate change intervention

**DOI:** 10.1038/s41598-017-12138-4

**Published:** 2017-09-20

**Authors:** Kevin D. Kroeger, Stephen Crooks, Serena Moseman-Valtierra, Jianwu Tang

**Affiliations:** 1 U.S. Geological Survey, Woods Hole Coastal & Marine Science Center, 384 Woods Hole Road, Woods Hole, MA 02543 USA; 2 Silvestrum Climate Associates, LLC, 150 Seminary Drive, 1E, Mill Valley, CA 94941 USA; 3 University of Rhode Island, Department of Biological Sciences, 120 Flagg Road, Kingston, RI 02881 USA; 4 Marine Biological Laboratory, Ecosystems Center, 7 MBL Street, Woods Hole, MA 02543 USA

## Abstract

Coastal wetlands are sites of rapid carbon (C) sequestration and contain large soil C stocks. Thus, there is increasing interest in those ecosystems as sites for anthropogenic greenhouse gas emission offset projects (sometimes referred to as “Blue Carbon”), through preservation of existing C stocks or creation of new wetlands to increase future sequestration. Here we show that in the globally-widespread occurrence of diked, impounded, drained and tidally-restricted salt marshes, substantial methane (CH_4_) and CO_2_ emission reductions can be achieved through restoration of disconnected saline tidal flows. Modeled climatic forcing indicates that tidal restoration to reduce emissions has a much greater impact per unit area than wetland creation or conservation to enhance sequestration. Given that GHG emissions in tidally-restricted, degraded wetlands are caused by human activity, they are anthropogenic emissions, and reducing them will have an effect on climate that is equivalent to reduced emission of an equal quantity of fossil fuel GHG. Thus, as a landuse-based climate change intervention, reducing CH_4_ emissions is an entirely distinct concept from biological C sequestration projects to enhance C storage in forest or wetland biomass or soil, and will not suffer from the non-permanence risk that stored C will be returned to the atmosphere.

## Introduction

Methane emissions from wetlands, predominantly from freshwater systems such as peatlands and tidal fresh and low salinity wetlands, comprise about 1/3 of global CH_4_ emissions from all sources^1^. Soil microbial respiration and low rates of methane oxidation in anaerobic, water-saturated soils result in substantial CH_4_ emissions in those freshwater settings. Depending on the timescale of analysis, climate warming due to CH_4_ emissions can partially or entirely offset climatic cooling due to C sequestration in freshwater wetland soil^2^. In contrast, in saline wetlands, including salt marshes, saline mangroves, and seagrass beds, CH_4_ emissions are typically minor because abundant sulfate ion in seawater limits microbial CH_4_ production and emission^3^. Thus, with high rates of net C storage and minor CH_4_ emission, saline wetlands generally have a strong cooling effect on climate^3–6^.

**Figure 1:**
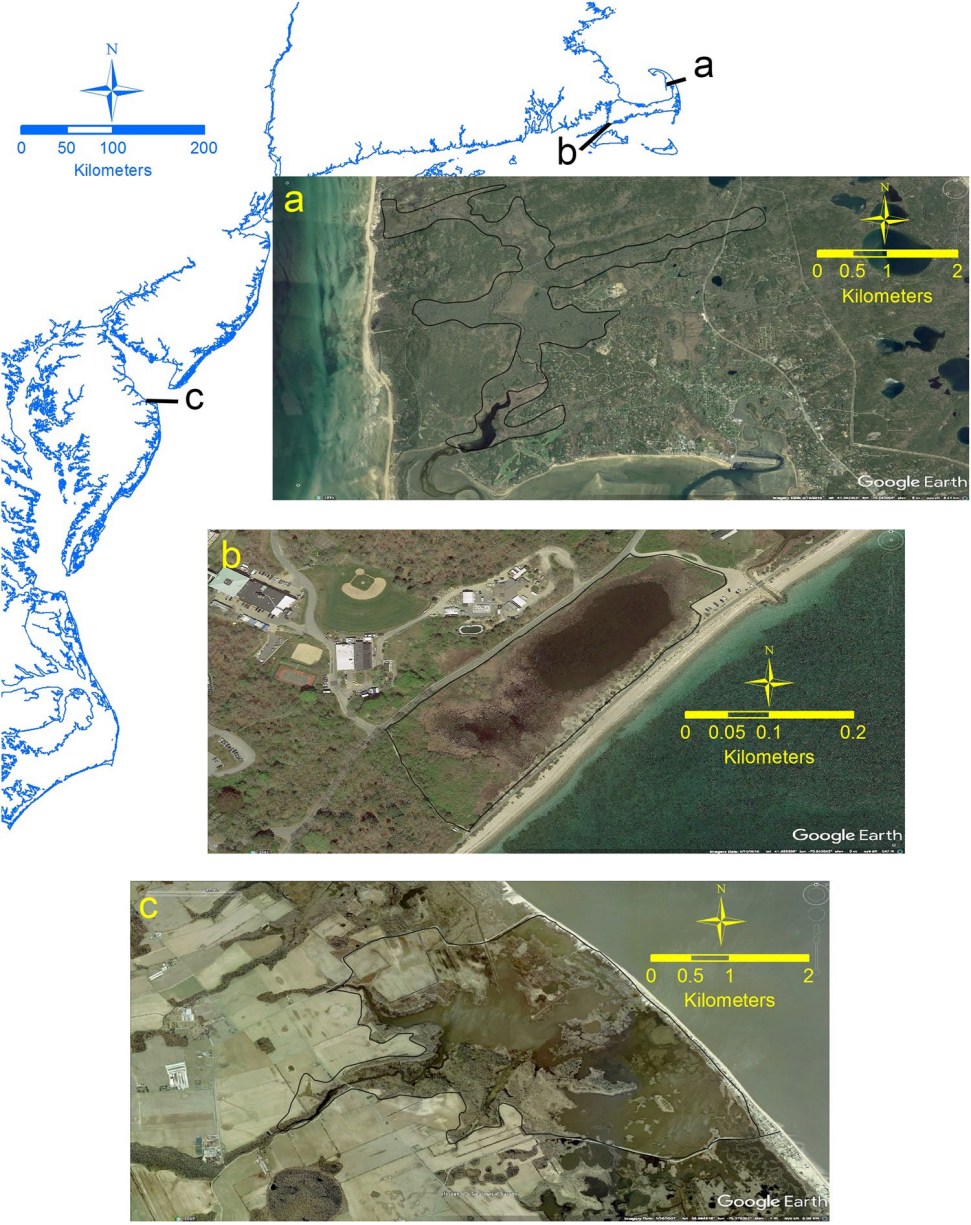
Satellite photographs of tidally-restricted wetlands on the U.S. Atlantic coast, demonstrating a range of histories, causes and sizes: (**a**) The Herring River basin within the National Park Service Cape Cod National Seashore, Massachusetts. The solid black outline indicates 4 km^2^ of former tidal salt marsh. The estuary and marsh were diked in the first decade of the 20^th^ century, in an unsuccessful attempt to control mosquitos. Portions of the basin are now^7^ drained marsh, and are characterized by acid-sulfate soil, loss of elevation, and colonization by native and non-native grass, shrub and trees. Other portions are impounded, freshened and colonized by native and non-native fresh and brackish wetland grasses. The wetland is proposed for tidal restoration, and pre-restoration carbon cycle and ecological data collections are underway. (**b**) An impoundment on ~0.05 km^2^ of former salt marsh on the south shore of Cape Cod, Massachusetts, caused by a 19^th^ century railroad that was subsequently converted to a bicycle path. Similar incidental impoundments are common and widespread along coastal transportation corridors. (**c**) The Prime Hook National Wildlife Refuge in the state of Delaware. The Refuge is a 41 km^2^ complex of managed wetland and open water. In response to repeated storm damage, plans were in development to upgrade the artificial berm that impounded 16 km^2^ of freshwater impoundment (https://www.fws.gov/refuge/prime_hook/). Prior to completion of the upgrades, Hurricane Sandy breached the artificial berm in 2012 and restored saline tidal flows to the impoundment. Subsequently, deliberate salt marsh restorations were undertaken. The inadvertent breach likely resulted in significant GHG emission reductions. Maps created by georeferencing each image from Google Earth (image source credits: (**a**) Google, Landsat/Copernicus, copyright DigitalGlobe; (**b**) Google, Landsat/Copernicus; (**c**) Google, USGS), base map and scale bars were added using ArcGIS for desktop version 10.3.1 (http://www.esri.com), and images arranged using Adobe Photoshop CC 2017 vers. 18.0.1 (http://www.adobe.com/products/photoshop.htm).

Commonly, however, tidal exchange of saline water between the coastal ocean and emergent, tidal wetlands, including salt marshes and mangroves (herein referred to as “tidal wetlands”), has been blocked or restricted by human activity, and those alterations can dramatically freshen and degrade the ecosystem^7,8^. Both drainage and impoundment of tidal wetlands have been practiced for a wide range of purposes during the past several centuries of human civilization^7–9^. Blockage or restriction of tidal flows, through installation of dikes or tide gates, is a common method to protect coastal infrastructure; to drain tidal wetlands for farming, mosquito control, and development; or to raise or manage water tables and reduce salinity for aquaculture, mosquito control, rice production, and wildfowl management. Inadvertent tidal restrictions also occur due to road, railroad and other infrastructure development, with affected wetlands landward of transportation corridors often becoming freshened and flooded due to retention of freshwater drainage from the watershed^8^. As a result of those many causes for complete or partial tidal restriction in tidal wetlands, inhabited and developed coastal landscapes typically contain a patchwork of unaltered tidal wetlands interspersed with drained, impounded, and partially restricted wetlands, ranging in size from <0.1 km^2^ to multiple km^2^ (Fig. 1). In many cases, if the alteration occurred decades or centuries ago, the parcels may no longer be recognized as former salt marsh by local residents or by the National Wetlands Inventory^10^, but rather as fresh or brackish ponds or wetlands where impounded, or, where drained, former wetland may appear as woodland, shrubland, developed land, or agricultural land.

Restriction or blockage of tidal flows dramatically alters wetlands as habitat for flora and fauna^8^, and further causes major changes in microbial and chemical processes in wetland soil and water^7,11^. Here we emphasize that those changes can also alter the direction and magnitude of greenhouse gas (GHG) exchange between the wetland and the atmosphere. The fate of soil C in wetlands, and emissions of CH_4_ and CO_2_, largely depend on water salinity and water table elevation relative to the soil surface, with temperature and plant productivity also having important influence. Under flooded or impounded conditions, soil becomes anaerobic, effectively to the soil surface. Under those conditions, if blockage of tidal flows has caused water salinity to decrease from >18 psu (~50% seawater) to <18, then a substantial increase in CH_4_ emission is expected to occur^9^. Salinity is a critical driver because seawater contains abundant sulfate ion, and where sulfate is abundant, sulfate reduction tends to outcompete methanogenesis as a metabolic pathway for microbial decomposition of organic matter. Additionally, sulfate can serve as the terminal electron acceptor in the oxidation of methane to carbon dioxide^12^, further reducing CH_4_ emissions under saline conditions. Though measurements are uncommon, CH_4_ emissions in inundated, low salinity portions of tidally-restricted wetlands are elevated^13,14^, and similar to the average of published rates for tidal wetlands at salinity <18 psu^3^. Drainage of both tidal and non-tidal wetlands, on the other hand, drains water from soil pore-spaces and exposes soil organic matter to air and associated oxygen, thereby promoting aerobic microbial respiration of C stocks that have accumulated over centuries to millennia, with CO_2_ as the primary product. Drainage-associated soil CO_2_ emissions are intense per unit area, and drained tidal wetlands^15^ and terrestrial peatlands^16^ are significant drivers of climate change. Drainage and restoration of terrestrial (non-tidal, freshwater) peatlands are not the focus of the present study, except as an informative comparison to GHG consequences of tidal restriction and restoration of tidal wetlands, since rewetting (re-flooding through restoration of natural hydrology) of terrestrial peatlands, to protect soil C stocks from ongoing respiration and loss as CO_2_ emissions, is considered to have great potential as a landuse-based climate intervention^17^.

**Figure 2:**
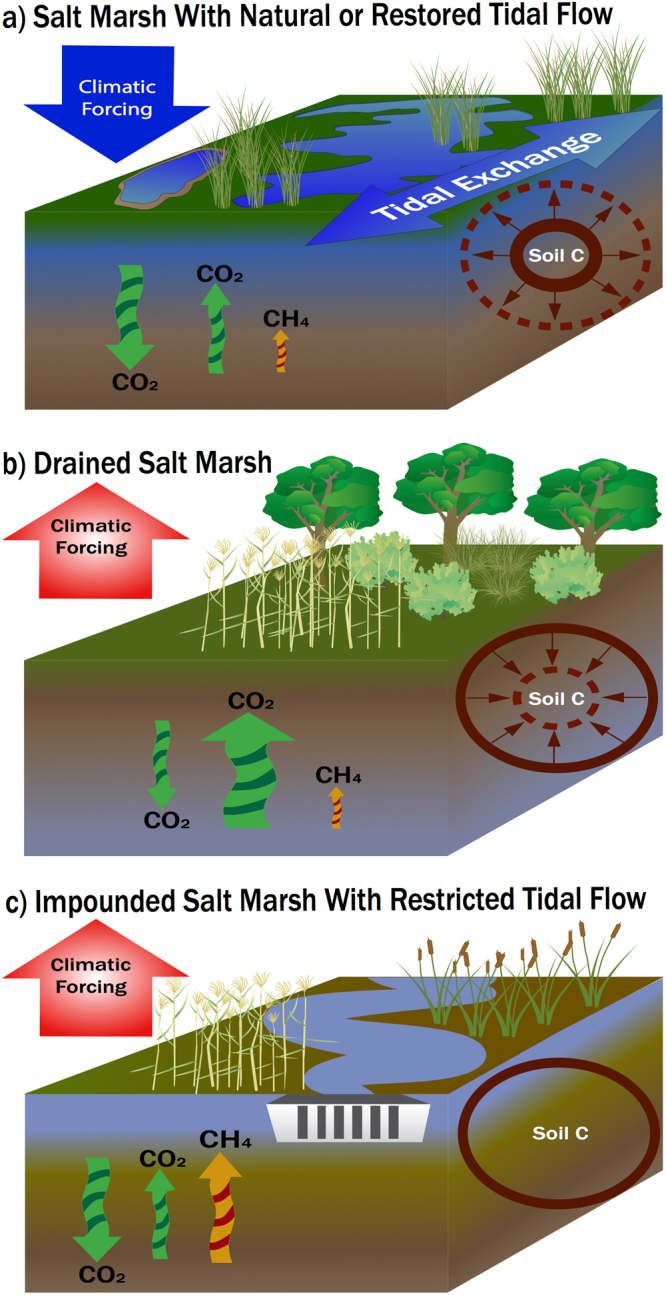
Conceptual model of carbon cycle processes and greenhouse gas flux changes in response to hydrological management in tidal wetlands. (**a**) In unaltered, or successfully restored, salt marsh, sulfate ion inhibits methane emission, and high rates of net CO_2_ uptake, soil C storage, and soil elevation gain occur. (**b**) Salt marsh drainage increases exposure of soil carbon stocks to oxygen, and results in a rapid rate of aerobic respiration to CO_2_, loss of carbon stock, and loss of elevation. (**c**) Impoundment commonly leads to freshening and increased water level, and those conditions are likely to cause an increase in methane emission. Effects of impoundment on soil carbon stocks and rate of soil carbon storage are not well-known, and herein rates are assumed to be similar to natural salt marsh. Diagrams created using Adobe Illustrator CC and arranged using Microsoft PowerPoint vers. 15.32. Diagram symbols were provided courtesy of the Integration and Application Network, University of Maryland Center for Environmental Science (ian.umces.edu/imagelibrary/).

**Figure 3:**
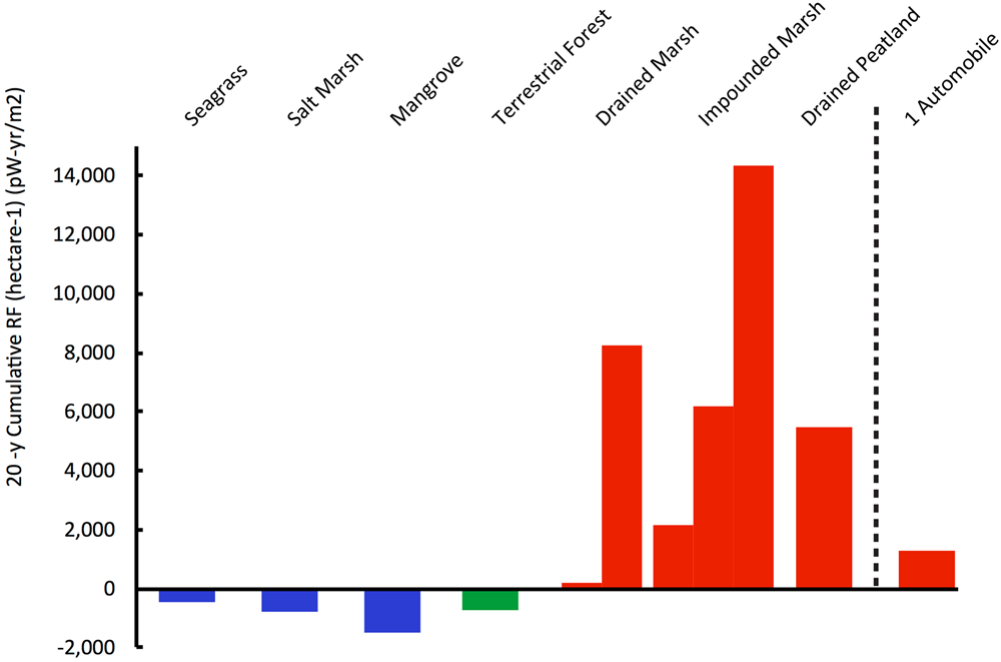
Twenty-year cumulative radiative forcing (picowatt-years per m^2^ of earth surface) due to net effect of carbon sequestration and methane emission in typical coastal wetlands and in hydrologically-altered wetlands. Radiative forcing is calculated per hectare (10^4^ m^2^) of wetland, with continuous emissions over a 20-year period, based on emission factors provided in Table 1. As described in the text, scenarios were considered based on two emission factors for carbon dioxide in drained marsh and three emission factors for methane in freshened or impounded wetlands. Those EF are shown here as separate bars. For context, emissions in wetlands are compared to terrestrial forest C sequestration and to emissions from an automobile. RF from an automobile was calculated based on twenty years of tailpipe emissions from one U.S. automobile^50^ at 4,690 kg CO_2_ y^−1^.

To examine the intensity of GHG emissions due to tidal restriction in salt marshes, as well as the potential for emissions reductions through tidal restoration, we modeled resulting changes over time in the cumulative mass of atmospheric CO_2_ and CH_4_ emitted or sequestered, and their radiative forcing (RF, a measure of the change in energy in the global atmosphere). The concepts around changes in soil C stocks and in GHG emissions with impoundment or drainage of salt marsh, and with restoration of tidal flows, are diagramed in Fig. 2. Here, “tidal restoration” describes removal or opening of dikes, tide gates, or under-sized culverts to renew tidal water exchange between the salt marsh and coastal ocean, and thus restore natural, or nearer to natural, water level and salinity. We first compared the intensity, per unit area, of cooling (net negative RF) in natural Blue Carbon ecosystems (seagrass beds, salt marshes and mangroves) to intensity of warming due to enhanced CH_4_ and CO_2_ emissions in impounded and drained salt marsh, respectively (Fig. 3). As further context, we also modeled: climatic cooling (negative RF) from net C storage in U.S. forests; warming (positive RF) from tailpipe emissions of a typical U.S. automobile; and warming (positive RF) from enhanced CO_2_ emissions in drained, terrestrial peatlands. We utilized RF calculations in this study to enable comparison of the climatic impact of CO_2_ and CH_4_ emissions or emissions reductions that are sustained over a period of decades to centuries. Use of standard global warming potentials (GWP) to compare the impact of different GHG is not appropriate in the context of sustained emissions that are characteristic of ecosystems and their management^4^. Rather, standard GWP calculations are intended as a policy tool based on analysis of the atmospheric perturbation lifetime of a discrete pulse of GHG emission. Standard GWP calculations underestimate the climatic impact of sustained CH_4_ emissions or emissions reductions relative to sustained changes in CO_2_ emissions.

In the present study, emission factors (EF), or typical CH_4_ and CO_2_ emission rates for each ecosystem type or condition, were derived from the literature and from IPCC^18^ tier 1 EF (Table 1). To represent the range and variability of CH_4_ emissions in low salinity tidal wetlands, and therefore expected range of emissions and EF in freshened, impounded wetlands, we modeled the time course of RF using three different CH_4_ EF based on the median and average of published emission rates^3^ at salinity <18, as well as the IPCC EF for low salinity tidal wetlands. The wide range in literature estimates for CH_4_ emissions in low salinity tidal wetlands is likely due to variations across sites in other drivers of emissions, including water level, plant productivity and temperature. Therefore, it is important to examine a range of EF for these low salinity wetlands, to represent the diversity of emissions. For CO_2_ EF in drained and rewetted terrestrial and tidal wetlands, we used IPCC Tier 1 EF from drained and rewetted inland organic soils and tidal wetlands. Note that there is uncertainty regarding the length of time over which the high rate of CO_2_ emission will occur following drainage. The duration will depend in part on the depth of drainage and whether further, deeper drainage occurs following initial drainage^19^, thereby exposing new soil C to oxidation. We therefore used two CO_2_ EF. EF1 assumed that drainage-associated respiration had gone to completion during decades to centuries of drainage, and current and future drainage-associated CO_2_ emission is zero. EF2 assumed that C stocks were not depleted, and CO_2_ emission continued over the time period of analysis.

**Table 1:** Methane and carbon dioxide emission rates utilized as inputs to model radiative forcing in a series of wetland settings and restoration scenarios.

Restoration Scenario		GHG Emission Factors Prior to, and Post-Restoration			
		CH_4_ prior (g C m^−2^ y^−1^)	CH_4_ post (g C m^−2^ y^−1^)	CO_2_ prior (g C m^−2^ y^−1^)	CO_2_ post^k^ (g C m^−2^ y^−1^)
1. Tidal restoration to: Flooded & freshened salt marsh	CH_4_ EF 1	8.4^a^	0.46^e^	−91^g^	−91^l^
	CH_4_ EF 2	19.4^b^	0.46	−91	−91
	CH_4_ EF 3	41.6^c^	0.46	−91	−91
2. Tidal restoration to: Drained salt marsh	CO_2_ EF 1	0.64^d^	0.46	0^h^	−91
	CO_2_ EF 2	0.64	0.46	790^i^	−91
3. Create salt marsh		0	0.46	0	−91
4. Create seagrass bed		0	0	0	−43^l^
5. Re-wet drained peatland		0.64	12.15^f^	517.5^j^	−15.5^m^
Setting		CH_4_ (g C m^−2^ y^−1^)		CO_2_ (g C m^−2^ y^−1^)	
6. Saline mangrove		0.46^e^		−162^l^	
7. Terrestrial forest				−69.6^n^	

^a^Poffenbarger *et al*.^3^, median of compiled data from wetlands with salinity <18.

^b^Hiraishi *et al*.^18^, Table 4.14.

^c^Poffenbarger *et al*.^3^, average of compiled data from wetlands with salinity <18.

^d^Hiraishi *et al*.^18^, Table 2.3, assumed equivalent to rate for drained inland peatlands.

^e^Poffenbarger *et al*.^3^, average of compiled data from wetlands with salinity >18.

^f^Hiraishi *et al*.^18^, Table 3.3, average rate for boreal and temperate peatlands.

^g^Assumed net C sequestration equivalent to saline wetland.

^h^Scenario in which soil has been drained for several decades, and major respiratory loss of soil C has gone to completion.

^i^Hiraishi *et al*.^18^, Table 4.13; Scenario in which soil has been drained for <30 years and major respiratory loss of soil C continues until restored.

^j^Hiraishi *et al*.^18^, Table 2.1, average of rates for boreal, temperate and tropical peatlands; Scenario in which major respiratory loss of soil C continues until restored.

^k^In all scenarios, assumed lag of 5 years, associated with ecosystem establishment, prior to initiation of new C sequestration in soil^34^.

^l^Hiraishi *et al*.^18^, Table 4.12.

^m^Hiraishi *et al*.^18^, Table 3.1, average of rates for boreal and temperate peatlands.

^n^EPA^51^, Tables 6–10 and 6–12, average rate of biomass, litter and soil C stock increase in U.S. forests, after correction to remove emissions from forest fires (Table 6–13).

## Results

### Radiative Forcing per Unit Area

Results indicate that climatic warming, or positive RF, due to tidal restriction (both impoundment and drainage) is large on a per unit area basis relative to the magnitude of cooling from C sequestration in forests and in unaltered coastal wetlands (Fig. 3). For instance, over a period of 20 years following initial alteration, CH_4_ emissions from an impounded and freshened salt marsh can result in a cumulative RF that is a factor of ~3 to 20 greater than the magnitude of climatic cooling due to net C stock increase in continental U.S. forests and to soil C sequestration in unaltered salt marsh, and a factor of 1.5 to 10 greater than sequestration in natural mangrove. Over those 20 years, CH_4_ emissions from one hectare of impounded wetland are equivalent to RF from 20 years of continuous tailpipe emissions of 1.7 to 6.3 automobiles (Fig. 3).

**Figure 4:**
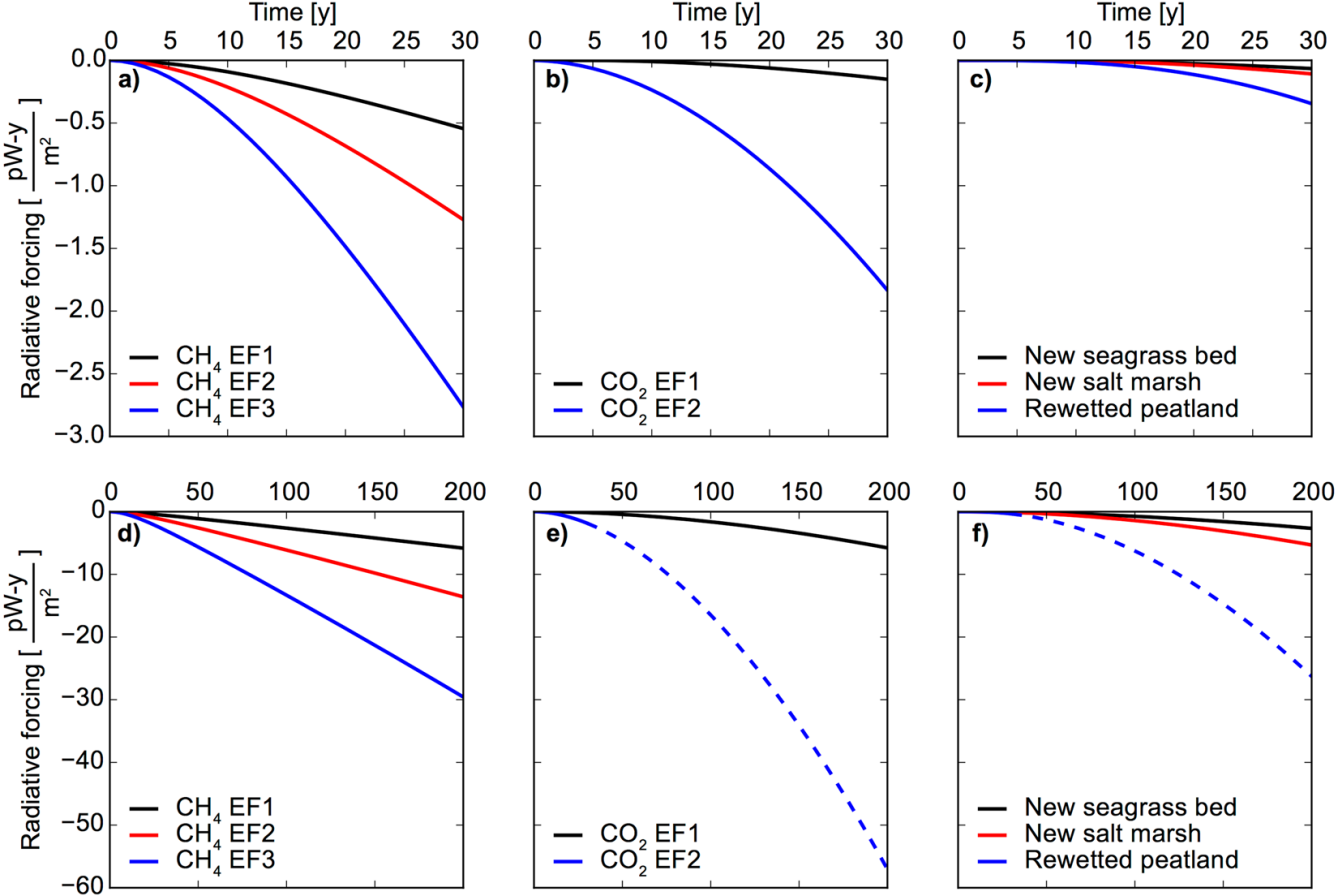
Time-course of cumulative radiative forcing modeled as the difference between emissions under scenarios of no action and of restoration (Table 1). Negative values indicate cumulative climatic cooling (picowatt-years per m^2^ of earth surface) per square meter of wetland restored, over a 30-year period (**a**,**b**,**c**) and a 200-year period (**d**,**e**,**f**). Methane emission reductions due to tidal restoration in impounded (flooded and freshened) former salt marsh (panels a and d) result in large reduction in RF, particularly on a timescale of decades to a century. Carbon dioxide emission reductions due to rewetting of drained salt marsh (**b**,**e**) and terrestrial peatland (**c**,**f**) also have relatively large climate benefit, but depend on assumptions regarding the period of time that the wetland would have been maintained in a continuously drained condition, and the period of time that the finite carbon stock in the drained soil would continue to respire (uncertainty regarding timeframe of benefit indicated by dashed lines; see text).

To quantify the potential for emissions reductions through wetland management, we calculated the time-course, over decades to centuries, of cooling (negative RF) predicted to occur due to CH_4_ and CO_2_ emissions reductions resulting from tidal restoration in tidally-restricted salt marsh, based on the cumulative difference between the contrasting management action and no action scenarios (Fig. 4). We compared climatic cooling due to tidal restoration to cooling from other wetland carbon management options, including creation of new salt marsh or sea grass beds as biological C sequestration projects, and to rewetting of terrestrial peatland to cease the high rate of CO_2_ emissions from drained peatland soils. In general, the tidal restorations in salt marshes, to restore natural salinity and water levels, will be dramatically more effective at cooling climate than other wetland-based climate change interventions (Fig. 4). The time course of cumulative RF resulting from reduction in the rate of CH_4_ production in previously flooded and freshened soils (Fig. 4a,d), and reduction in CO_2_ production in previously drained soils (Fig. 4b,e) indicate sizeable, rapid and sustained climatic cooling. Despite the high rate of C sequestration in salt marshes and mangroves, building and planting of new marsh, mangrove or seagrass bed, provides relatively modest climatic cooling (Fig. 4c,f). Avoided CO_2_ emissions from rewetting of drained, terrestrial peatlands (Fig. 4c,f) can be substantial, but are partially offset by resumption of CH_4_ emissions in soil rewetted with fresh water^20^ (Table 1).

### Geography of Tidally-Restricted Wetlands and Scale of Emissions

There is substantial potential for GHG reductions based on the intensity of emissions, and on the areal extent of tidally-restricted wetlands in developed landscapes. Tidally-restricted wetlands are widespread throughout the inhabited world (e.g.^7,8,13–15,18–31^). Infrastructure and activities that alter wetland and coastal hydrology have been closely associated with intensity of human development and economic activity in terrestrial and marine portions of the coastal landscape^31,32^, and therefore are expected to increase in the future, particularly in portions of the world that are undergoing rapid development^23,31^. Quantitative geographical data on tidally-restricted wetlands are limited, and indeed the global area of tidal wetlands as a whole is not well-constrained^15^. Thus, it is not possible to estimate global scale GHG emissions due to restrictions. As an indication of scale, we used mapped and areal data along portions of the U.S. Atlantic coast on managed impoundments and on incidental impoundments associated with transportation infrastructure. The analysis resulted in an estimate that those tidal restrictions have caused a reduction in salinity, and enhancement of CH_4_ emissions, in approximately 27% of tidal wetlands on the U.S. Atlantic coast (Table 2). The impoundment and freshening result in enhanced CH_4_ emissions in the range of 28,000 to 145,000 tonnes (t) CH_4_ y^−1^. It is worth emphasizing once again that these are sustained emissions, and thus RF due to those rates of continuous emission over a 20-year period are equivalent to 20 years of continuous emissions from 0.6 to 3.1 million automobiles (see Methods). Note that this is an estimate of CH_4_ emissions alone, and does not consider changes in C sequestration rate in soil, nor loss of existing soil C stocks and enhanced soil CO_2_ emissions, due to changes in water level and salinity. Further, the area and emissions calculations consider only the effect of freshening and impoundment of tidal wetlands, and do not include area or emissions of tidal wetlands that have been drained, unintentionally nor intentionally, for purposes such as agriculture or land development.

**Table 2:** Geography of tidally restricted wetlands on the Atlantic coast of the United States.

Location	Tidal Wetland in Study Area (km^2^)	Wetland Area Affected (km^2^)	Fraction of wetland area affected (%)
**Transportation-Related Restrictions:**			
Southern Maine^24^	32	9	28
New Hampshire^24^	26	5	20
Massachusetts^46^	212	58	27
North Shore^47^	113	6	5
Cape Cod^48^	70	20	28
Buzzards Bay^49^		33	
Rhode Island^52^	16	11	70
**Total Transportation**	**286**	**84**	**29**
**Impounded for Waterfowl or Mosquito Management:**			
N. Carolina^9^	643	21	3
S. Carolina^9^	2,041	285	14
Georgia^9^	1,590	32	2
Florida (Atlantic)^9^	778	143	18
**Total Impounded for Wildlife Management**	**5**,**053**	**482**	**10**
**Total impounded tidal wetlands U.S. Atlantic coast**, **Transportation** + **Wildlife**	**9**,**710** ^10^	**3**,**787**	**39**
**Total area impounded and freshened:**			
U.S. Atlantic coast	**9**,**710**	2,650	27

Available published data were compiled on surface areas of managed wetland impoundments and incidental, full or partial impoundment resulting from transportation infrastructure. Tidally restricted wetland areas were used as a sample to estimate restricted wetland area as a percentage of total tidal wetland area. Percentages were then extrapolated to the areas without data. Based on salinity data^9^ and vegetation as an indicator of salinity^49^, it was estimated that 70% of the impounded wetlands were freshened as a result. (see Methods).

## Discussion

### Key features of avoided wetland methane emissions

The results of this study indicate that, despite a high rate of C sequestration in coastal wetlands, in many cases tidal restoration in salt marshes will have dramatically greater potential per unit area as a climate change intervention than the other examined ecosystem management actions. Coupled with the common, widespread occurrence of tidal restrictions, there is significant potential for GHG emissions reductions through tidal restoration in salt marshes. Though we did not address tidal restriction and restoration in mangroves, many of the processes and rates presented for salt marshes would be expected to be similar in mangroves. We further note that avoiding tidal restrictions with future coastal development would have similar and perhaps greater benefits in terms of avoided GHG emissions than the restoration scenarios examined in this study. Here we discuss several features of tidal restoration, and particularly avoided methane emissions, that highlight further advantages relative to enhanced CO_2_ sequestration in other landuse-based climate change interventions, due to key aspects that result in rapid, substantial, and sustained reduction in RF:

First, CH_4_ has a radiative efficiency, or instantaneous heat absorption capacity, that is a factor of ~73 greater than that of CO_2_, and a factor of ~94 greater with consideration of indirect effects of CH_4_ on atmospheric chemistry^33^. Second, reductions in both CH_4_ and CO_2_ emissions are soil microbial responses, and thus, though more data are needed, onset following restoration of natural water level and salinity is likely to be rapid^14,21^ relative to development of restored C storage capacity following full ecosystem recovery or development in biological sequestration projects^34^. In the present study, ecosystem recovery was represented as a lag of 5 years prior to onset of the full rate of C sequestration in salt marsh soil (Table 1, note *k*). Thus, climatic cooling due to avoided CH_4_ emissions is relatively large on short timescales. In contrast, in projects that promote biological C sequestration in wetland soil, or similarly in forest biomass, accrual of climate change mitigation benefits is a process of gradual accumulation on a timescale of centuries (see “New salt marsh” curve in Fig. 4c and f), due to both the gradual rate of ecosystem development following restoration, and to the modest radiative efficiency of CO_2_, coupled with a long atmospheric perturbation lifetime. As a result, at 15 years post restoration, for instance, negative RF (cooling) due to CH_4_ reduction (Fig. 4a) was a factor of 12 to 59 greater than that due to CO_2_ sequestration in new salt marsh (Fig. 4c).

Third, in the absence of restoration, enhanced CH_4_ emissions in an impounded and freshened wetland can be expected to continue indefinitely, due to a continuous supply of new organic matter substrate for methanogenesis from ongoing primary production^35^. Thus, although CH_4_ is considered to be a short-lived climate pollutant, with an atmospheric perturbation lifetime of only 12.4 years, restoration can avoid a long-term, sustained emission, and the cumulative mass of avoided CH_4_ and magnitude of climatic cooling will continue to increase indefinitely through time (Fig. 4d). In contrast, although avoidance of CO_2_ emissions by tidal restoration in drained salt marshes can have an RF in the first few decades that is as great as avoided CH_4_ in freshened marshes, the duration and ultimate cumulative magnitude of enhanced CO_2_ emissions in a drained wetland is limited by the mass of the C stock contained in soil above the level of drainage. Therefore, where wetlands have been drained for decades to centuries, the benefit of restoration may be relatively minor (Fig. 4b emission factor 1). Where pre-restoration CO_2_ respiration is rapid, re-wetting will be of substantial benefit (Fig. 4b emission factor 2), but the anticipated duration of that benefit will be uncertain (Fig. 4e). Based on IPCC Tier 1 marsh soil C stocks and respiration rates in drained marshes, the stock in the top meter of soil would be consumed within approximately 3 decades following drainage^18^.

A fourth critical feature of wetland tidal restoration as a climate change intervention is that the CH_4_ emissions thus avoided will have what can be referred to as “inherent permanence”: following tidal restoration and onset of reduced methane emissions, the emissions thus avoided cannot be cancelled, even if emissions were to resume in the future due to a change in ecosystem status. For instance, if a tidal restriction were re-established 30 years after restoration, emissions would resume, but the biosphere and atmosphere would maintain for extended time a deficit of heat and of GHG equivalent to 30 years of reduced emissions. Further, since GHG emissions from tidally-restricted wetlands are caused by human activity, they are anthropogenic emissions, and reducing anthropogenic wetland emissions will have an effect on climate that is equivalent to reduced emission of an equal quantity of fossil fuel GHG. Therefore, avoiding CH_4_ emissions as a landuse-based climate change intervention is a distinct concept from biological C sequestration projects to enhance C storage in forest or wetland biomass or soil. Biological C sequestration accumulates climate benefit at a relatively slow rate, and the C sink must persist for a century or more to have significant impact on climate (see Fig. 4f, new salt marsh example). During that time, there is a risk that the stored C will be rapidly returned to the atmosphere, through processes such as fire or ecosystem degradation and organic matter decomposition. As a result, the monetary and climate change mitigation value of biological C sequestration projects must be discounted in C markets and in trading programs to account for the “non-permanence risk” that stored C will be returned to the atmosphere^36,37^. To be clear, this discussion is not intended to suggest that temporary reductions in CH_4_ emissions will achieve effective management of climate change, but rather to highlight a distinction between avoided emissions and C sequestration.

### Implications for GHG emission inventories and reduction programs

Tidal restoration to avoid CH_4_ emissions is a relatively new concept for Blue Carbon management, and warrants consideration within GHG markets and emission offset programs, national and local-level efforts to reduce emissions, and national GHG inventories based on IPCC guidance. Within international climate change treaty negotiations there has been increasing emphasis on co-benefits accruing from GHG mitigation actions that involve improved landscape management, including forests, soils and potentially coastal and marine ecosystems. In 2013, the International Panel on Climate Change released the 2013 Supplement to the 2006 IPCC Guidelines for National GHG Inventories: Wetlands^18^, to guide accounting of emissions and management of wetland soils. The Supplement includes guidance on CO_2_ emissions for drained wetlands, but does not consider GHG emissions from impoundments in general, nor freshened or impounded wetlands due to tidal restriction. The U.S. has recently completed the first national inventory worldwide to include coastal wetlands in a National GHG Inventory, and the UN Framework Convention on Climate Change has asked countries to provide feedback on experiences with application of the Supplement, to inform future expansion of the accounting guidance where science is available. The analysis presented here indicates that tidally restricted wetlands meet the primary criteria for inventoried ecosystems in that they are managed landscapes, with emissions and sinks of substantial magnitude and rate of change. If other countries ultimately follow suit, then inclusion of those emissions in the U.S. Inventory will promote widespread recognition and management of the issue, and justify development of CH_4_ EF for tidal restrictions and impoundments in IPCC guidance for GHG inventories. Note that since ecological restoration through tidal reconnection has been a widespread practice in some parts of the world, restorations that were conducted since the baseline year, typically 1990, may represent considerable, but as yet unrecognized, emissions reductions.

Wetland C and GHG management can also play a role as an additional method for reducing regional, state and local GHG emissions, including through regulatory or voluntary carbon markets or cap & trade programs. Verified Carbon Standard (VCS) has developed a Methodology for crediting tidal wetland carbon offset projects, designed for both voluntary and regulatory C markets worldwide, and within that methodology there is accommodation for avoided CH_4_ projects as a creditable activity^37^. The implications of the results presented in the present study may support continued development of concepts related to crediting of climate change mitigation through avoided CH_4_ emissions in wetlands, to reflect the distinctions between avoided CH_4_ and C sequestration projects:

First, at present, in the VCS methodology, there must be reasonable expectation that a wetlands-based, emissions offset project will have a lifetime of at least 100 years. The mandated lifetime is in response to the concept that in projects that promote C sequestration in soil or biomass, the sequestered C must be kept out of the atmosphere for an extended time to have significant climate change mitigation value. However, in the context of avoided CH_4_ emissions, the 100-year time frame is arbitrary, since such offset projects do not rely on storage of C in a soil or biomass reservoir, and they accrue considerable, long-term and quantifiable mitigation benefit on timeframes of decades. Given that the persistence of any project for 100 years is difficult to assure, a shortened minimum lifetime for avoided CH_4_ projects could increase utilization of tidal wetlands as offset projects.

Second, the application of a 100-year CH_4_ global warming potential of 21 to calculate carbon dioxide equivalents (CO_2_e) of avoided CH_4_ emissions underestimates the climate change mitigation value of the avoided CH_4_ to a substantial degree. As an example, if we were to apply that GWP value in a CO_2_e approach to scenarios 1 and 3 in Table 1 (tidal restoration in salt marsh to avoid CH_4_ emissions vs. creation and planting of new salt marsh to enhance C sequestration), the range of CH_4_ emission factors would result in a calculated GHG reduction benefit for avoided CH_4_ (scenario 1) that is similar to the C sequestration benefit in scenario 3 (scenario 1 = 0.7 to 3.5 times the value of scenario 3; not shown). The ratio would remain constant for the 100-year lifetime of the projects. That result is in contrast to the more nuanced calculation of change in radiative forcing that we noted previously in the manuscript, in which reduction in RF (cooling) due to CH_4_ reduction (Fig. 4a) was a factor of 12 to 59 greater than RF reduction due to CO_2_ sequestration in new salt marsh (Fig. 4c), at 15 years post restoration. Even at 100 years post restoration, the benefit of avoided CH_4_ emissions (scenario 1) remains a factor of 3 to 11 greater (Fig. 4d and f). As noted previously, a standard GWP approach is inappropriate for sustained emissions or sinks that are typical of ecosystems^4^. A 100-year GWP that is calculated based on simulation of a single pulse of CH_4_, with an atmospheric lifetime of 12.4 years, integrates 12 years of heat retention across 100 years of impact, and ignores the fact that with sustained emissions a pool of CH_4_ would be maintained in the atmosphere indefinitely, rather than for just 12 years.

There is ongoing debate regarding the value, in terms of addressing climate change, of reducing CH_4_ emissions vs CO_2_ emissions, and the debate is relevant to selection of a GWP value for CH_4_, and crediting timeframe, in C markets and offsets. Though there is not a simple answer, it is clear that aggressive reduction in emissions of CH_4_ and other short-lived climate pollutants (SLCP) can reduce the rate of increase in global temperature during the current century, and can reduce peak temperature if coupled with rapid elimination of CO_2_ emissions^38^. Further, it has been shown that, although CH_4_ is a short-lived climate pollutant, during the time that it resides in the atmosphere, a portion of the heat that it traps within the biosphere will be retained in oceans and contribute to sea level rise for several centuries^39^. However, if actions to reduce emissions of SLCP result in delayed reductions in CO_2_ emissions, then higher peak temperatures will result^38^. Therefore, society is likely to benefit most from simultaneous, but separate, efforts to reduce both CO_2_ and SLCP emissions, to manage both short-term and long-term climate change. Utilization of offsets and emissions trading programs therefore requires relative evaluation of pollutants with distinct interactions with climate. Solutions might be to limit maximum utilization of offsets in C markets, as is done in California’s Cap and Trade Program^40^, or to develop separate reduction and trading programs for CO_2_ and SLCP. For instance, the state of Massachusetts has specific emissions reduction goals for natural gas transmission^41^, New Zealand has separate targets for methane reductions in their Intended Nationally Determined Contribution^42^, and many national and subnational governments have programs to reduce methane emissions from agricultural sources^43^. As described in the present study, degraded tidal wetlands can be significant sources of both CO_2_ and CH_4_, while intact wetlands are generally a strong CO_2_ sink. Thus, wetland management can contribute to management of both short and long-lived climate pollutants.

Finally, beyond C markets and national GHG inventories, awareness of anthropogenic emissions in tidally-restricted wetlands, and the potential for mitigation through ecosystem restoration, may be of significant value to land management agencies with wetlands, tidal impoundments, and coastal infrastructure under their purview, such as the U.S. Fish and Wildlife Service, the National Park Service, local and state government entities, and private land owners. In wetlands that are intentionally managed, in many cases opening tidal restrictions would be prohibited by ongoing important land uses, or where restoration would put developed land at risk of flooding. But, increasingly, decisions are being forced, by accelerating sea level rise and increasing storm intensity, regarding whether to spend resources to preserve and upgrade aging dikes and tide gates that maintain wetlands in drained or impounded condition (for example see Fig. 1c, Prime Hook NWR). Management decisions and objectives are based in part on anticipated changes in the value of ecosystem services to society, including coastal protection, quality and type of habitat, recreational utility, and others. Reduction in GHG emissions has significant value to society, and can be highlighted as a benefit, regardless of whether the benefit is monetized. Even where opening tidal restrictions is contraindicated, there still may be potential for management of water levels and salinity at targeted times of the year, specifically for the purpose of reducing GHG emissions and promoting C sequestration, while maintaining utility of the landscape for those other uses or protecting infrastructure from flooding.

In other circumstances, the decisions may be less complicated in terms of tradeoffs of services, such as where tidal restriction is due to inadvertent, transportation-related obstruction of flow, and thus there is no current beneficial use of the impaired wetland (for example, see Fig. 1b). In some cases, ecosystem restoration through opening of tidal restrictions can be consistent with coastal resilience planning, since exposure to tidal flow will tend to promote resumption of natural accretion of wetland elevation in response to sea level rise, resulting in enhanced protection of the infrastructure landward of the wetland.

There is significant potential for C and GHG management in coastal wetlands, but continued advances are needed on the scientific basis for policy frameworks and for quantification of emission factors in response to management actions. There has been particularly little study of changes in soil and biomass C stocks, and of GHG fluxes, in response to tidal restriction and tidal restoration in salt marshes and mangroves. Further, in all areas of the world the state of knowledge is poor regarding the geographic distribution of tidal wetlands, of salinity within those wetlands, of occurrence of tidally-restricted salt marshes and mangroves, and of the potential for tidal restoration. The findings presented here therefore suggest a research agenda to study GHG emissions and the fate of carbon stocks in natural, restricted and restored wetlands, across geographic settings, as well as mapping occurrence and characteristics of tidal wetlands and tidally-restricted wetlands.

## Methods

Radiative Forcing—To calculate radiative forcing, we modeled the time-course of change in the atmospheric inv entory of each GHG per square meter of wetland, based on emission rate and on rate of destruction or removal from the atmosphere. Methane was modeled as a single reservoir with atmospheric perturbation lifetime of 12.4 years, and first order removal due to oxidation to CO_2_. RF from management of methane emissions includes forcing from the resulting changes in both the pool of atmospheric CH_4_ and CO_2_ produced through CH_4_ oxidation. We calculated the mass of CH_4_ in the atmosphere, per square meter of wetland at time *t*, according to Neubauer and Megonigal^4^ eq. 1:

1 $${M}_{C{H}_{4}-C,(t)}={F}_{C{H}_{4}-C}dt+[{M}_{C{H}_{4}-C,(t-1)}\times {e}^{(-dt/{{\rm T}}_{C{H}_{4}})}],$$

where ${M}_{C{H}_{4}-C}$ is the mass of atmospheric CH_4_ (g C m^−2^), ${F}_{C{H}_{4}-C}$ is the emission factor (g C m^−2^ y^−1^), *dt* is the time step (0.2 y), and T_CH4_ is the atmospheric perturbation lifetime of CH_4_.

Mass of atmospheric CO_2_ was modeled based on a synthesis of models, utilizing four non-interacting reservoirs with distribution of emissions among the reservoirs and atmospheric perturbation lifetimes in the reservoirs as in Joos *et al*.^44^, Table 5 and IRF model within that publication. The mass of CO_2_ in the atmosphere was calculated as:

2 $${M}_{C{O}_{2}-C,(t)}=\sum _{i=1}^{4}\,{f}_{i}({F}_{C{O}_{2}-C}dt+[{M}_{C{O}_{2}-C\_i,(t-1)}\times {e}^{(-dt/{{\rm T}}_{C{O}_{2}\_i})}],$$

where *f*_*i*_ is the fraction of CO_2_ emissions distributed to the *i* ^*th*^ reservoir, and other terms are as defined for the CH_4_ model.

In both models, the instantaneous RF was calculated at each time step as the product of the mass of each gas in the atmosphere and its radiative efficiency^33^ (1.75 × 10^−15^ W m^−2^ (kg CO_2_)^−1^ and 1.28 × 10^−13^ W m^−2^ (kg CH_4_)^−1^ with CH_4_ radiative efficiency adjusted by a factor of 1.65 to account for indirect effects^45^. Cumulative RF is the sum of instantaneous RF over a given time period. N_2_O emissions were not considered due to insufficient data.

Geography—Large scale, geographic data on tidal restrictions are rare, but a large number of examples and case studies of restriction and restoration occur within the literature. As an indication of scale, approximately 50% of pre-development tidal saltmarsh area in the U.S. has been destroyed by human activities, with impoundment and drainage as prominent causes^23^, and a quarter of remaining coastal wetlands in the state of Massachusetts, for example, is landward of nearly 600 transportation-related tidal restrictions^46–49^. To estimate the scale of tidally restricted wetlands area and the proportion of existing wetlands that are restricted, we compiled published information on the occurrence along the U.S. Atlantic coast of managed wetland impoundments and incidental, full or partial impoundments caused by transportation infrastructure (Table 2). In the northeast U.S., wetland and impoundment areas landward of primarily transportation-related restrictions comprise 29% of total tidal wetland. In the southeast U.S. 10% of total tidal wetland area is diked and impounded for waterfowl and mosquito management. The tidal wetlands in the regions and states represented in the analysis comprise 55% of the total tidal wetland on the U.S. Atlantic coast, and thus are expected to be approximations of the entire coast and may be similar to many developed coastal regions elsewhere. Based on the assumption that similar levels of transportation-related and diked restrictions occur throughout the U.S. east coast, we estimate that 39% of total tidal wetland area on the U.S. Atlantic coast, ~3,800 km^2^, is above tidal restrictions or blockages. Montague *et al*.^23^, reported that 87% of the southeast U.S. mosquito and waterfowl impoundments included in Table 2 had salinity less than 16 psu. Thus, they are expected to be significant methane sources. It is not known what proportions of restricted wetlands related to transportation infrastructure are in freshened, drained, or relatively unaltered condition. In Massachusetts 70% of tidally restricted wetland area is described as freshwater marsh or fresh shrub, with 52% of the vegetated area colonized by invasive *Phragmites australis*, while the remainder remains brackish to saline marsh^48,49^. In general, where purposeful actions have not been taken to drain tidally-restricted wetlands, such as is the case with incidental restrictions caused by transportation infrastructure, flooding and freshening are the most likely result. Thus, we can approximate that 70% of restricted wetland area compiled here, or 2,650 km^2^, is in a flooded/freshened condition. To calculate minimum and maximum estimates of CH_4_ emission from those wetlands, we multiplied the area by the minimum and maximum annual emissions rates of CH_4_ from impounded wetlands (Table 1, Scenario 1, EF1 and EF3 pre-restoration minus post-restoration). For context, the annual emission rates from 2,650 km^2^ of freshened wetlands were compared to average annual tailpipe emissions from a U.S. automobile^41^, at 4,690 kg CO_2_ y^−1^, with the comparison calculated as cumulative RF over a 20-y period of continuous emissions at those rates. The resulting anthropogenic wetland emission estimate was 28,000 to 145,000 tonnes CH_4_ y^−1^, with RF equivalent to 20 years of continuous emissions from 0.6 to 3.1 million automobiles (not shown).

### Data availability

The data that support the analyses presented are included in tables and in cited references. Results of radiative forcing calculations are available upon request from the lead author.
